# Treatment strategies in colorectal cancer patients with initially unresectable liver-only metastases, a study protocol of the randomised phase 3 CAIRO5 study of the Dutch Colorectal Cancer Group (DCCG)

**DOI:** 10.1186/s12885-015-1323-9

**Published:** 2015-05-06

**Authors:** Joost Huiskens, Thomas M van Gulik, Krijn P van Lienden, Marc RW Engelbrecht, Gerrit A Meijer, Nicole CT van Grieken, Jonne Schriek, Astrid Keijser, Linda Mol, I Quintus Molenaar, Cornelis Verhoef, Koert P de Jong, Kees HC Dejong, Geert Kazemier, Theo M Ruers, Johanus HW de Wilt, Harm van Tinteren, Cornelis JA Punt

**Affiliations:** 1 Department of Surgery, Academic Medical Centre, Meibergdreef 9, 1105 AZ Amsterdam, The Netherlands; 2 Department of Radiology, Academic Medical Centre, Amsterdam, the Netherlands; 3 Department of Pathology, VU University Medical Centre, Amsterdam, the Netherlands; 4 Netherlands Comprehensive Cancer Organisation, Utrecht, the Netherlands; 5 Department of Surgery, UMCU, Utrecht, the Netherlands; 6 Department of Surgery, Erasmus MC Cancer Institute, Rotterdam, the Netherlands; 7 Department of Surgery, University Medical Center Groningen, University of Groningen, Groningen, the Netherlands; 8 Department of Surgery, MUMC, Maastricht, the Netherlands; 9 Department of Surgery, VU University Medical Centre, Amsterdam, the Netherlands; 10 Department of Surgery, Netherlands Cancer Institute, Amsterdam, the Netherlands; 11 Department of Surgery, Radboud University Medical Center, Nijmegen, the Netherlands; 12 Department of Biostatistics, Netherlands Cancer Institute, Amsterdam, the Netherlands; 13 Department of Medical Oncology, Academic Medical Centre, Amsterdam, the Netherlands

**Keywords:** Unresectable colorectal liver metastases, Treatment strategies

## Abstract

**Background:**

Colorectal cancer patients with unresectable liver-only metastases may be cured after downsizing of metastases by neoadjuvant systemic therapy. However, the optimal neoadjuvant induction regimen has not been defined, and the lack of consensus on criteria for (un)resectability complicates the interpretation of published results.

**Methods/design:**

CAIRO5 is a multicentre, randomised, phase 3 clinical study. Colorectal cancer patients with initially unresectable liver-only metastases are eligible, and will not be selected for potential resectability. The (un)resectability status is prospectively assessed by a central panel consisting of at least one radiologist and three liver surgeons, according to predefined criteria. Tumours of included patients will be tested for *RAS* mutation status. Patients with *RAS* wild type tumours will be treated with doublet chemotherapy (FOLFOX or FOLFIRI) and randomised between the addition of either bevacizumab or panitumumab, and patients with *RAS* mutant tumours will be randomised between doublet chemotherapy (FOLFOX or FOLFIRI) plus bevacizumab or triple chemotherapy (FOLFOXIRI) plus bevacizumab. Radiological evaluation to assess conversion to resectability will be performed by the central panel, at an interval of two months.

The primary study endpoint is median progression-free survival. Secondary endpoints are the R0/1 resection rate, median overall survival, response rate, toxicity, pathological response of resected lesions, postoperative morbidity, and correlation of baseline and follow-up evaluation with respect to outcomes by the central panel.

**Discussion:**

CAIRO5 is a prospective multicentre trial that investigates the optimal systemic induction therapy for patients with initially unresectable, liver-only colorectal cancer metastases.

**Trial registration:**

CAIRO 5 is registered at European Clinical Trials Database (EudraCT) (2013-005435-24).

CAIRO 5 is registered at ClinicalTrials.gov: NCT02162563, June 10, 2014.

## Background

Approximately 50% of patients with colorectal cancer (CRC) will develop metastases, either at presentation or during follow-up. Colorectal cancer disseminates predominantly to the liver [1]. The 5-year overall survival rates in patients with metastatic CRC have increased over the past decades due to the availability of more effective drugs and the increased use of resection of metastases, and is currently around 20% [2]. Complete resection of metastases offers the only chance for cure, however a minority of patients (approx. 20%) present with resectable metastases. Evidence for the benefit of neoadjuvant chemotherapy with the objective to improve resectability rates was already established in 1996, at which time it was shown that initially unresectable metastases could become resectable (further defined as secondary surgery) after downsizing by chemotherapy [3]. Currently there is consensus that combination chemotherapy should be part of this neoadjuvant regimen, however there is no consensus regarding the selection of targeted therapy.

### Secondary liver resections after neoadjuvant systemic treatment

Data from a single institution by Adam et al. [4] have shown that of 1104 patients with metastases confined to the liver, 12.5% of patients became eligible for secondary surgery, and that these patients had a 5-year survival rate of 33%. The benefit of primary or secondary surgery has not been evaluated in prospective randomised studies. However, given the consistent data from published series, there is little doubt that a complete resection (primary or secondary) of liver metastases prolongs survival. Indeed, in the liver survey database the survival benefits of secondary surgery are close to those of primary surgery, and better than for systemic therapy alone [5].

A major problem in interpretation of the results of these studies is the lack of consensus on the criteria for resectability, as has been shown in the CELIM study [6]. This complicates the interpretation of the results from studies involving patients with unresectable liver-only metastases, and even more of the results on secondary resection rates as reported from retrospective subgroup analyses from phase 3 studies in unselected metastatic colorectal cancer patients.

### Choice of chemotherapy regimen in neoadjuvant treatment

Randomised phase 3 studies have clearly shown that combination chemotherapy with a fluoropyrimidine plus irinotecan or oxaliplatin produces higher response rates compared with fluoropyrimidine monotherapy [7]. Therefore combination chemotherapy should be used when downsizing of metastases is the primary objective.

Studies on triple chemotherapy (5FU + oxaliplatin + irinotecan, FOLFOXIRI) have shown high response rates in phase-2 studies, but conflicting results on its survival benefit in two phase-3 studies [8-10]. However, retrospective analysis of both phase 3 studies showed that the rate of secondary resections was increased, from 5 (12%) to 14 (36%) and from 6 (4%) to 14 (10%), respectively. It should be noted that secondary resections were not a prospective objective of these studies.

### Neoadjuvant treatment with chemotherapy plus either anti-EGFR antibodies or bevacizumab

Given the slightly higher response rates of chemotherapy plus anti- EGFR antibodies (cetuximab, panitumumab) compared to chemotherapy plus bevacizumab in the first-line treatment of metastatic CRC patients, the use of cetuximab or panitumumab has been advocated in patients with potentially resectable liver metastases. However, an increase in the response rate has also been shown in some (but not all) phase 3 studies by the addition of bevacizumab to chemotherapy. Also high secondary R0 resection rates were obtained in phase 2 studies with chemotherapy plus bevacizumab [11,12]. Data from 2 randomised trials of a head-to-head comparison between bevacizumab and anti-EGFR therapy, both in combination with chemotherapy, do not show a significant difference in response rate and progression-free survival [13,14]. Also preliminary results of the larger CALGB 80405 trial do not show a significant difference in overall survival [15]. The results of the TRIBE study [16] showed a significant benefit in response rate for FOLFOXIRI + bevacizumab versus FOLFIRI + bevacizumab (65% versus 53%, respectively). However, this did not translate into an increased rate of secondary resections (15% versus 12%, and in patients with liver-only metastases 32% versus 28%, respectively).

Furthermore, the use of RECIST criteria in the evaluation of the effect of targeted therapies has been questioned, and data are accumulating that morphological criteria rather than RECIST criteria should be used to assess the efficacy of bevacizumab treatment [17,18].

As a backbone for the use of targeted therapies, currently no preferred chemotherapy regimen prevails. The benefit of bevacizumab and anti-EGFR antibodies has been shown in combination with both irinotecan- and oxaliplatin-containing regimens [19]. A head-to-head comparison of irinotecan- and oxaliplatin-containing regimens in combination with cetuximab has shown comparable results in patients with unresectable liver-only metastases [6]. However, the use of capecitabine in combination with anti-EGFR therapy is being discouraged [20].

### Selection of patients for anti-EGFR therapy

Since the initial observation that *KRAS* mutation (exon 2, 3 en 4) is a negative predictive factor for anti-EGFR therapy [21], much effort has been made to further optimize patient selection for this therapy. Recently, the negative predictive value of *RAS* (*KRAS exon* 2,3 en4 and *NRAS* exon 2 and 3) mutations were confirmed [22,23]. *BRAF* mutation was shown to be prognostic, but not predictive [24,25].

## Methods/design

The objective of the CAIRO5 study is to provide prospectively derived data on neoadjuvant systemic treatment strategies in patients with initially unresectable colorectal cancer liver metastases while using uniform and transparent criteria for unresectability. Given the lack of a predictive model that allows the selection of patients in whom a secondary resection may be achieved, the inclusion is not limited to patients with potentially resectable metastases and we plan to include all patients with unresectable, liver-only metastases.

Patients with *RAS* wild type tumours are randomised between bevacizumab and panitumumab in combination with a two-drug combination chemotherapy (5-fluorouracil plus either irinotecan, FOLFIRI, or oxaliplatin, FOLFOX, according to choice of the local investigator). Although panitumumab and cetuximab were shown equally effective in patients with *KRAS* wild type tumours [26], we selected panitumumab as anti-EGFR antibody given the more mature data for panitumumb in relation to *RAS* mutation status. Patients with *RAS* mutated tumours will be randomised between FOLFOX/FOLFIRI (choice of investigator) plus bevacizumab and triple chemotherapy (FOLFOXIRI) plus bevacizumab.

An innovative aspect of CAIRO5 is the prospective assessment of unresectability status and evaluation of treatment by a central panel of radiologists and liver surgeons, according to predefined and transparant criteria.

### Objectives and hypotheses

The primary objective of this study in CRC patients with initially unresectable liver-only metastases is to compare the median progression-free survival (PFS) between the two treatment strategies in each of the two patient cohorts (*RAS* wild type and *RAS* mutant tumors, respectively). In patients with *RAS* wild type tumours it is hypothesized that FOLFOX or FOLFIRI + panitumumab will improve PFS as compared to FOLFOX or FOLFIRI + bevacizumab. In patients with *RAS* mutant tumours it is hypothesized that FOLFOXIRI + bevacizumab will improve PFS as compared to FOLFOX or FOLFIRI + bevacizumab.

Secondary objectives are to assess the secondary R0/1 resection rate, median overall survival, response rate, toxicity, pathological response in resected lesions, postoperative morbidity, and correlation of baseline and follow-up evaluation by the panel with outcome. Translational research will be performed on predictive and prognostic biomarkers and imaging methods.

### Study design

The study is designed as a randomised phase 3 trial. For each candidate patient, a panel of at least 3 liver surgeons and one radiologist will evaluate the baseline CT scan of abdomen and liver for resectability or unresectability of liver metastases (see Panel procedure and evaluation).

Potentially eligible patients will be registered after informed consent has been obtained. Once eligibility has been confirmed, including the unresectabilty status of liver metastases as defined by the central panel, patients will be randomised and *KRAS (exon 2, 3 and 4), NRAS (exon 2 and 3) and BRAF* mutation status will be assessed using TSACP MiSeq analysis [27]. Patients with *RAS* wild type tumours are being randomised between doublet chemotherapy plus either bevacizumab or panitumumab. Patients with *RAS* mutated tumours are being randomised between doublet chemotherapy plus bevacizumab or triple chemotherapy plus bevacizumab. RAS wild type patients and RAS mutant patients will be randomised independently in a 1:1 ratio. Randomisation will be done using ALEA software (ALEA®, FormsVision, Abcoude, the Netherlands).

**Figure 1:**
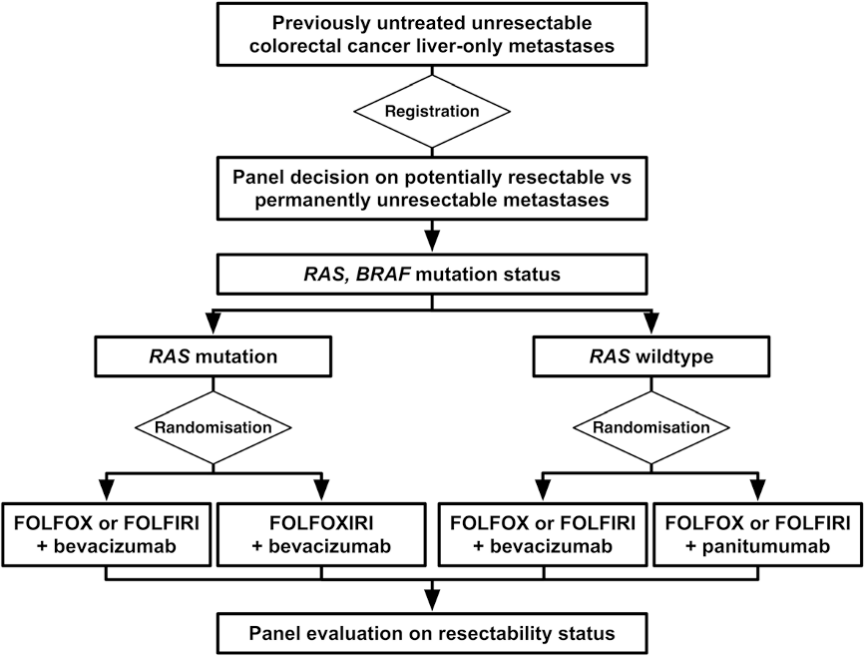
Study design CAIRO5.

Patients will be stratified for potential resectability of liver metastases (yes versus no, according to the central panel), serum LDH (normal versus abnormal), and treatment centre. Patients with *RAS* wild type tumours will also be stratified for *BRAF* mutation status (wild type versus mutated) and use of irinotecan- versus oxaliplatin-containing regimen. The flowchart of the study is shown in Figure 1.

### Study population

Patients who meet the following inclusion criteria are eligible for participation in this trial: histological proof of colorectal cancer, previously untreated and unresectable metastases confined to the liver (as assessed by the central panel) according to CT scan obtained less than 2 weeks prior to registration, adequate tumour tissue available for assessment of *RAS* and *BRAF* mutation status, WHO performance status 0-1 (Karnofsky performance status ≥ 70), age ≥ 18 years, no contraindications for liver surgery, resectable primary tumour if still *in situ*, adequate organ functions, life expectancy over 12 weeks, expected adequacy of follow-up, and written informed consent.

Exclusion criteria are: previous systemic treatment for metastatic disease, extrahepatic metastases, with the exception of small (≤1 cm) extrahepatic lesions that are not suspicious of metastases, unresectable primary tumour, serious comorbidity or any other condition preventing the safe administration of study treatment (including both systemic treatment and surgery), major cardiovascular event within 12 months before randomisation, uncontrolled hypertension, or unsatisfactory blood pressure control with ≥3 antihypertensive drugs, previous adjuvant treatment unless completed ≥ 6 months prior to randomisation, previous surgery for metastatic disease, previous intolerance of study drugs in the adjuvant setting, pregnant or lactating women, second primary malignancy within the past 5 years with the exception of adequately treated in situ carcinoma of any organ or basal cell carcinoma of the skin, any concomitant experimental treatment.

### Panel procedure and evaluation

A central panel consisting of at least 3 liver surgeons and one radiologists will review the CT scans for resectability status prior to randomisation and at first evaluation (after 4 treatment cycles), and, if deemed necessary, at second evaluation (after 8 treatment cycles), and at third evaluation (after 12 treatment cycles). The central panel is blinded for the treatment arm. Any further review will take place according to panel decision.

By general consensus among Dutch hepatic surgeons, and for purpose of transparency and uniformity, unresectability *at baseline* for this study is defined as the expected failure of achieving a complete (R0) resection of all lesions in one single surgical procedure (i.e. excluding 2-stage resections, use of portal vein embolization) by surgical resection only (i.e. excluding the use of RFA or other surgical methods), leaving a minimum remnant liver volume of 25-30% in normal livers, and 35-40% in compromised livers (fibrosis, cirrhosis or steatosis).

Once a patient has entered the study following these criteria, the central panel will evaluate resectability of liver metastases after every 4 treatment cycles (now also allowing the use of preoperative portal vein embolization and the combination with local ablative techniques such as RFA, or of a two-stage resection). The decision of resectability will be made by the central panel by majority vote. The chairman of the panel will coordinate the voting process and confirm final decision of the panel. Secondary resection should include all lesions as demonstrated at baseline imaging, however, when lesions have disappeared under treatment and are not detectable during the surgical procedure, these will be left in situ. The decision to perform secondary resection by laparoscopic or by open procedure is left to the discretion of the performing surgeon.

Patients’ images will be uploaded in a software program specially designed to share patient imaging in a safe and privacy-respecting environment. (ALEA®, FormsVision, Abcoude, the Netherlands).

### Study treatment: systemic therapy

Patients will be treated according to the assigned treatment regimen. All systemic treatment regimens are administered according to standard practice, and all cycles have a length of 2 weeks. The choice between FOLFIRI and FOLFOX is at the discretion of the local investigator and may be selected on a per patient basis.

The assigned treatment will be continued for at least 6 months (12 cycles) or until progression of disease, unacceptable toxicity, or patient refusal. If after 6 months the liver metastases are still not resectable it is highly unlikely that resectability will be achieved at all. These patients have liver metastases that remain unresectable after induction systemic therapy, however without progression of disease. They should continue with the targeted drug in combination with chemotherapy, but the chemotherapy should be continued as maintenance treatment with 5FU/LV alone. The targeted drug should not be replaced by any other targeted drug during first-line treatment prior to disease progression.

Treatment after first progression is not part of the study, however recommended strategies can be found in the study protocol.

In patients who become resectable and undergo secondary surgery of their liver metastases, the total duration of preoperative and postoperative treatment together should be 6 months, with the chemotherapy schedule being continued postoperatively according to the preoperative schedule. However, given the lack of benefit of adding a targeted drug to chemotherapy in the adjuvant setting of stage III colon cancer [28-30] as well as of resected liver metastases [31], the targeted drug will not be continued after surgery.

### Study statistics, sample size, planned analyses

The study is designed as a randomised phase 3 trial with progression free survival (PFS) as its primary endpoint. Two hypotheses will be tested simultaneously:

- in patients with *RAS* wild type tumours it is hypothesized that FOLFOX or FOLFIRI + panitumumab will improve PFS as compared to FOLFOX or FOLFIRI + bevacizumab.
- in patients with *RAS* mutant tumours it is hypothesized that FOLFOXIRI + bevacizumab will improve PFS as compared to FOLFOX or FOLFIRI + bevacizumab.

Given recent literature, it is expected that approximately 45% of the patients will have *RAS* (*KRAS* and *NRAS*) wild type tumours while 55% will have *RAS* mutated tumours.

The median PFS in patients with *RAS* wild type and *RAS* mutant tumours is estimated to be 10 months. The treatment is assumed to reduce the hazard rate for PFS by 30%. To detect such an improvement in PFS with 80% power and a two-sided logrank test at 5%, 247 events need to be observed. This requires an inclusion of approximately 640 patients, which are expected to be accrued in 4 years.

For the primary endpoint of PFS two interim analyses and a final analysis will be performed, equally spaced based on the number of events (approximately at one-third, two-third) of the way through the trial. At the interim analysis both futility and efficacy will be considered. The trial may be discontinued in either subgroup (RAS wild type and RAS mutated patients) when the treatment is very efficacious, but the trial may also be discontinued early in either subgroup if the new treatment is unlikely to show superiority to control based on the interim analysis.

Analysis of the primary endpoint will be based on the ‘intention-to-treat’ population. PFS by treatment arm will be calculated and depicted by means of the Kaplan Meier technique and will be compared using the (stratified) logrank test. Hazard ratios and 95% confidence intervals will be calculated with a (stratified) cox-proportional hazard analysis.

### Quality

Data management will be centrally and locally provided by the clinical research department of the Comprehensive Cancer Center in the Netherlands (IKNL).

This study will be monitored based on the recommendations as described in the brochure “Kwaliteitsborging mensgebonden onderzoek 2.0” published October 2012 by the Dutch Federation of University Medical Centres (NFU). Independent qualified monitors, local and central oncology data managers of IKNL clinical research department, will monitor the trial.

### Safety

In accordance to section 10, subsection 1, of the W.M.O. (Wet Medisch-wetenschappelijk Onderzoek met mensen), the investigator will inform the subjects and the reviewing accredited Medical Ethical Committee if anything occurs, on the basis of which it appears that the disadvantages of participation may be significantly greater than was foreseen in the research proposal. It is mandatory to record and report all serious adverse event (SAEs). The local investigators are responsible for reporting SAEs. All SAEs must be reported within 24 hours. The DCCG as the initiator is responsible for SAE assessment and reporting to the authorities in accordance with all requirements of the Dutch law. The DCCG has delegated these responsibilities to the principal investigator of this study. The sponsor will submit, once a year throughout the clinical trial, a safety report to the accredited Medical Ethical Committee, competent authority, and competent authorities of the concerned Member States.

In the CAIRO5 a Data Safety Monitoring Board (DSMB) is established to perform ongoing safety surveillance and to perform interim analyses on the safety data. This committee is an independent committee. The advice(s) of the DSMB will only be sent to the sponsor of the study. Should the sponsor decide not to fully implement the advice of the DSMB, the sponsor will send the advice to the reviewing METC, including a note to substantiate why (part of) the advice of the DSMB will not be followed.

### Ethics

This study will be conducted in accordance to the standards of Good Clinical Practice, in agreement with the Declaration of Helsinki (latest amendment), Dutch law in general and with the W.M.O. in particular.

The study has been approved by the medical ethical committee of the Academic Medical Centre Amsterdam, The Netherlands.

## Discussion

Secondary resection of liver metastases offers the only chance for cure in patients with initially unresectable, liver-only metastases. However data on secondary resection rates of initially unresectable colorectal cancer liver metastases are difficult to interpret. Most of these data are derived from retrospective studies, without data on outcome.

There are no data from prospective studies with transparent and uniform criteria for staging and resectability in patients with initially unresectable liver-only metastases. In the past, resectability of colorectal liver metastases has been based on tumour characteristics in the absence of extra-hepatic disease, such as the number of metastases, bilobar distribution, size of the largest metastasis and synchronicity. With improved treatment results and strategies these criteria have been modified. Currently, patients are selected on the basis of feasibility of achieving R0 resection with preservation of sufficient remnant liver to support metabolic liver function. Most surgeons will rely on a minimum of 25-30% of remnant liver, while maintaining adequate portal venous and hepatic arterial perfusion, hepatic venous drainage and biliary drainage.

Furthermore, based on the currently available data there is no outright preference for the use of either bevacizumab or anti-EGFR antibodies in combination with chemotherapy in patients with *(K)RAS* wild type tumours in whom secondary resection of metastases is the primary objective. Although its results are promising, triple chemotherapy with FOLFOXIRI has not shown to be outright superior in this respect to doublet regimens with FOLFOX or FOLFIRI.

In view of the considerations above, we elected to use clear-cut criteria for unresectability in the CAIRO5 study. Although by no means we consider these as the most optimal criteria, the transparent and reproducible nature of these criteria do allow to select a homogeneous patient population in terms of liver involvement, which subsequently facilitates the interpretation of our data. This is further supported by the use of a central panel that prospectively evaluates the status of liver metastases according to these criteria in all patients. We expect that the results of the CAIRO5 trial will contribute to define the optimal strategy in patients with initially unresectable, liver-only colorectal cancer metastases. The study is open for accrual as of July 2014.
